# Innovative Resource-Saving Equipment for Safflower Processing to Improve Oil Quality

**DOI:** 10.3390/foods14091596

**Published:** 2025-04-30

**Authors:** Maigul Mursalykova, Gulnara Kokayeva, Mukhtarbek Kakimov, Bożena Gajdzik, Radosław Wolniak, Damian Dzienniak, Michał Bembenek

**Affiliations:** 1 Department of Technological Equipment, Shakarim University of Semey City, Semey 071412, Kazakhstan; mursalykovamaigul@gmail.com; 2 Department of Technological Machinery and Equipment, S. Seifullin Kazakh Agro Technical Research University, Astana 010011, Kazakhstan; 3 Department of Technology of Food and Processing Industries, S. Seifullin Kazakh Agro Technical Research University, Astana 010011, Kazakhstan; muhtarbek@mail.ru; 4 Department of Industrial Informatics, Faculty of Materials Engineering, Silesian University of Technology, 44-100 Gliwice, Poland; bozena.gajdzik@polsl.pl; 5 Department of Economics and Informatics, Faculty of Organization and Management, Silesian University of Technology, 44-100 Gliwice, Poland; 6 Department of Manufacturing Systems, Faculty of Mechanical Engineering and Robotics, AGH University of Krakow, A. Mickiewicza 30, 30-059 Krakow, Poland; ddamian@agh.edu.pl (D.D.); bembenek@agh.edu.pl (M.B.)

**Keywords:** safflower oil production, sustainable oil production, oil quality enhancement, combined grinding and pressing, resource-efficient equipment, small and medium enterprises, physicochemical properties, fatty acid composition, optimal processing parameters, biologically valuable product

## Abstract

This study presents an innovative, resource-efficient apparatus for safflower oil production, designed for small- and medium-sized enterprises. The developed equipment integrates grinding and pressing into a single operation, optimizing extraction efficiency while reducing operational costs and processing time. A comparative analysis of the physicochemical and fatty acid profiles of safflower oil produced using traditional and combined grinding-pressing methods confirmed the superiority of the new approach. Optimal process parameters were identified: pressing time (*τ* = 3.41 min) and degree of grinding (*i* = 0.25), which resulted in higher oil yield and improved product quality. Multiple regression and sensitivity analyses revealed that temperature is the most significant factor positively influencing oil yield, whereas excessive grinding intensity and pressure negatively impact extraction efficiency. The results of the multiple regression analysis demonstrated that pressing time had a statistically significant impact on oil yield (*p* < 0.05). Although temperature was identified as the most influential factor overall, the significance of pressing time indicates that this variable also contributes meaningfully to the extraction efficiency. The regression model revealed a nonlinear (quadratic) relationship between pressing time and oil yield, suggesting that there is an optimal pressing duration. Beyond this optimal point, further increases in pressing time may result in a decline in yield due to over-compression or the release of undesired compounds. Interestingly, pressing time was found to have no significant effect on oil yield, suggesting that optimizing temperature and pressure is more critical for enhancing efficiency. The developed apparatus not only enhances oil quality, particularly its fatty acid composition, but also offers a scalable, sustainable solution for improving safflower oil production. These findings highlight the feasibility of integrating grinding and pressing, paving the way for advancements in cost-effective, high-yield oil extraction technologies.

## 1. Introduction

Safflower, an ancient crop belonging to the complex-flower family (*Asteraceae*), which includes artichoke, chicory, and sunflower, is cultivated widely across the globe. This versatile plant serves multiple purposes: its vibrant petals are used for food dyes, flavorings, and colorants, as well as in traditional medicine; its seeds are processed into vegetable oils and bird feed; and its leaves are utilized as livestock fodder [1,2].

Safflower oil is a premium-quality product rich in two key fatty acids, oleic and linoleic acids, which together comprise approximately 90% of its total fatty acid content [3,4,5]. The crop finds extensive application in medicine, pharmaceuticals, and food production [6,7]. Known for its resilience, safflower readily adapts to diverse growing conditions and does not require specialized sowing areas.

In recent years, global interest in safflower cultivation has surged, with total production reaching 718,161 metric tons [8]. Kazakhstan has emerged as a significant contributor, producing 174,900 metric tons annually, accounting for 24% of the global safflower output, which places the country among the world’s leading producers [9].

However, Kazakhstan has yet to establish itself as a major exporter of finished safflower oil. Addressing this challenge requires increasing the visibility of safflower oil, positioning it as a high-value finished product for export rather than merely a raw material, and enhancing its appeal to international consumers.

Pressing is one of the primary methods of vegetable oil production. Over the past several decades, extensive research conducted both in Kazakhstan and internationally has led to significant advancements in pressing efficiency, primarily through process intensification [10,11,12,13].

Despite these advancements, there is limited information in scientific literature on the performance of screw presses specifically designed for safflower oil extraction, and even less on machines that integrate combined processes. For oilseeds such as rapeseed [14], flax [15], sesame [16], sunflower [17,18,19], walnuts, pistachios [20,21], and safflower seeds, oil extraction is typically performed in separate units for crushing and pressing. This separation results in increased raw material losses during inter-operational transport, higher labor costs, and greater logistical complexity.

To address these challenges, integrating multiple processes into a single apparatus offers a promising solution. By combining crushing and pressing within a unified system, it becomes possible to reduce the number of operations, streamline production, and improve overall efficiency. Specifically, optimizing the pressing process with carefully balanced speed and pressure parameters can: intensify oil separation, minimize raw material losses, lower labor and production costs, reduce the physical footprint of production facilities, increase oil yield, enhance the nutritional value and density of the product for storage and transport, and broaden the potential applications of the final product [22].

The primary goal of this study is to identify optimal methods for safflower oil production at small- and medium-sized enterprises. This involves establishing the scientific, theoretical, and experimental foundations necessary to improve the pressing process through the integration of combined processes.

## 2. Materials and Methods

### 2.1. Crushing and Squeezing of Oil

The experiments utilized seeds of the safflower variety *Ahram*, with an initial moisture content of 12% and an average initial seed particle size of of 8–10 mm in length and 4–4.5 mm in width, which is widely cultivated in Kazakhstan owing to its high yield, resistance to diseases, and adaptability to diverse climatic conditions. This variety is predominantly grown in the Aktobe, Almaty, Zhambyl, Kyzylorda, South Kazakhstan, and Akmola regions.

**Figure 1:**
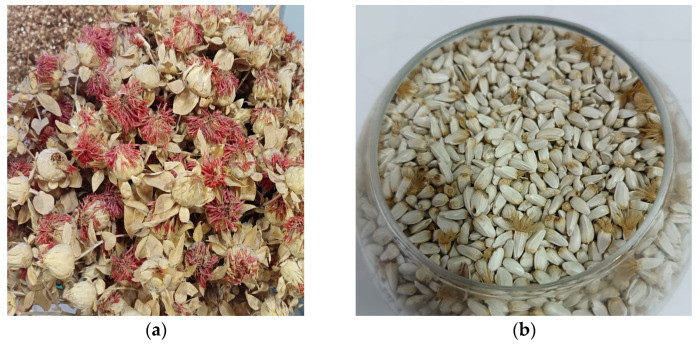
Flowers (**a**) and seeds (**b**) of safflower.

The fat content of *Ahram* seeds in their dry state is 37–38%, which is 13.8% higher than for the standard variety. Additionally, the average yield of *Ahram* is 9–10.5 centner/ha, which represents a 30% increase compared with the previously released variety [23,24]. Figure 1 presents safflower flowers and seeds.

Currently, traditional oil pressing methods using various screw oil presses are widely employed for both preliminary and final extraction of vegetable oil. These presses share similar working components and follow a common operational scheme [25,26,27,28,29,30].

The primary working elements of a screw oil press include the zeer cylinder and screw shaft. The process yields two final products: pressed vegetable oil and oil cake. As the screw shaft rotates within the zeer cylinder—a drum composed of slats (zeers) with small gaps—the material is transported from the loading zone to the unloading zone.

Compression occurs as the free volume of the coils decreases due to a reduction in screw pitch and an increase in the core diameter of the shaft from the beginning to the end of the screw. This compression generates pressure within the screw, effectively extracting oil from the seeds. The oil then flows through the gaps in the zeer cylinder and is collected in the oil pan. Meanwhile, the remaining pressed cake moves through the zeer cylinder and encounters a regulating mechanism that controls the thickness of the cake fraction at the oil press outlet.

As part of the research on safflower oil production at Shakarim University in Semey, the laboratory for “Improvement of Technique and Technology of Food Products” developed experimental equipment designed to combine the grinding and pressing processes into a single apparatus. This innovation is aimed at intensification of the pressing process and is protected by the patent for invention № 17072 and for utility model № 7977 of the Republic of Kazakhstan [31,32].

**Figure 2:**
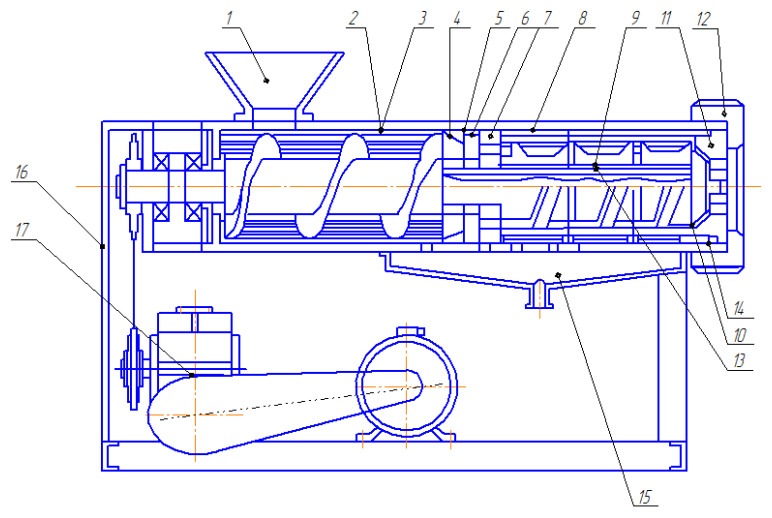
The scheme of the experimental press used in the tests: 1—hopper; 2—body; 3—auger shaft; 4—receiving plate; 5—ring; 6—rotary blade; 7—grinding plate; 8—grain cylinder; 9—screw winding; 10, 12—nut; 11—conical grating plate; 13—key; 14—spring; 15—tray; 16—welded frame; 17—drive unit [33].

The experimental press, illustrated in Figure 2, features several key components: a drive unit (17), a body (2), a hopper (1), and a tray (15) for oil collection, all mounted on a welded frame (16). The casing is divided into two main sections:

- The first section, which includes the auger shaft (3), a receiver (4), a grinding plate (7), a rotary blade (6), and a chopping mechanism composed of a ring positioned above the blade;
- The second section, which houses the screw winding (9) and intermediate ring links that make up the pressing mechanism, as well as a zeer cylinder with longitudinal holes and slots for oil extraction.

The grinding mechanism and screw windings of the pressing links are integrated into the auger shaft (3) and secured with a nut (10). At the outlet of the cylindrical stand, a conical grating plate (11) and fixing nut (12) are positioned, with adjustable springs (14) placed between them to regulate pressure.

**Figure 3:**
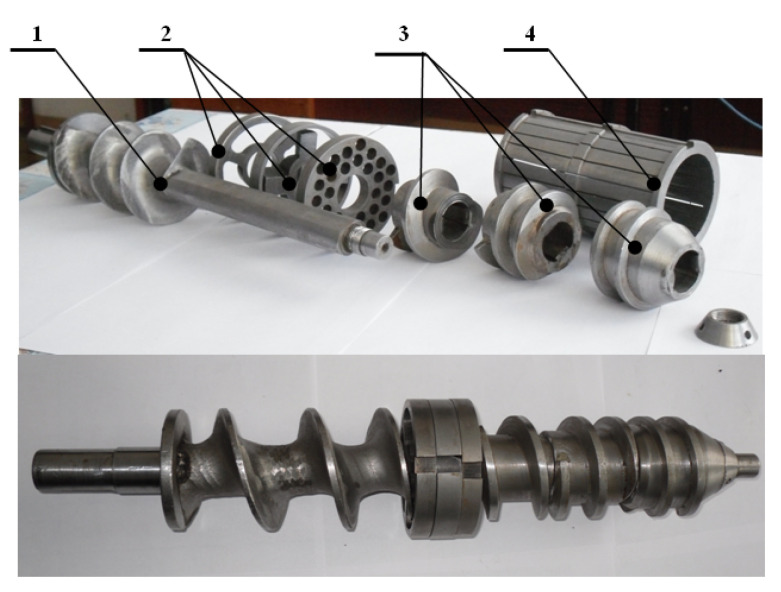
Main working components of the equipment: 1—conveying screw; 2—shredding mechanisms; 3—pressing mechanisms; 4—zeer cylinder [33].

**Figure 4:**
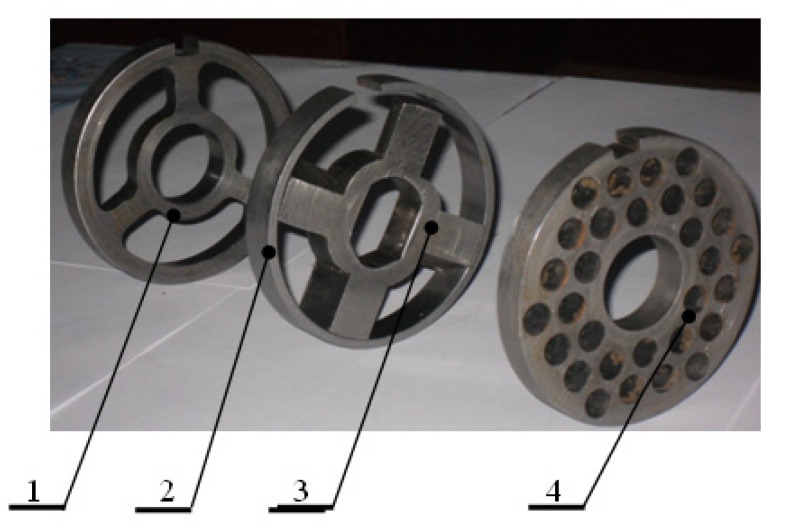
Set of shredding mechanisms: 1—receiving grid; 2—ring; 3—blade; 4—fine grinding grid [33,34].

**Figure 5:**
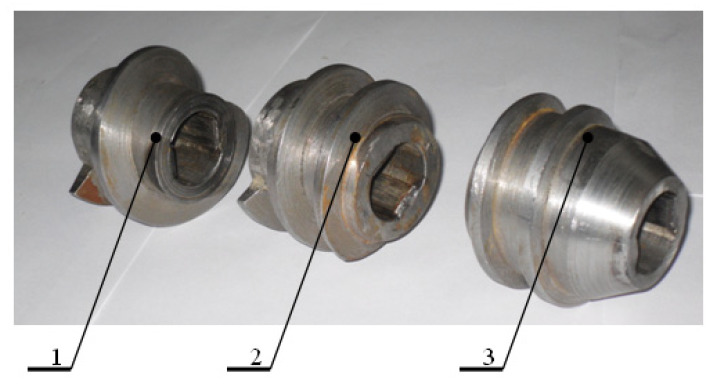
Set of pressing mechanisms: 1—primary pressing winding; 2—intermediate pressing winding; 3—final pressing winding [33].

The main working components of the equipment are presented in Figure 3. A detailed view of the shredding mechanisms is shown in Figure 4, whereas the pressing mechanisms are presented in Figure 5.

The press drive system comprises an electric motor, a worm gearbox, and a V-belt transmission. The worm gearbox is linked to the tail support via a chain transmission, which ensures smooth power transfer. The press shafts are securely connected to the tail support mounted on a sturdy welded frame. During the research, the rotational speed of the shafts was adjusted by replacing the pulleys in the V-belt transmission.

During the experiment, the measurement of key technological parameters during pressing was conducted using verified and calibrated instruments. To measure pressure in the pre-matrix chamber, a system of pressure transducer sensors was installed, enabling precise monitoring of pressure levels in the pre-matrix area of the press, with a maximum capacity of 25 MPa and an accuracy of ±1 kPa. The temperature distribution within the processed material in different sections of the press was measured using a universal device equipped with temperature sensors, ensuring reliable data collection.

The press operates as follows: A 5 kg batch of the initial product is fed into the system and transported by longitudinal grooves inside the cylindrical rack, assisted by the supporting screw. Simultaneously, the product undergoes crushing in the crushing mechanism before entering the pressing chamber.

In the pressing chamber, the material is compressed by the pressing screw, forcing the oil to separate and drain through longitudinal and cylindrical holes on the inner surface of the cylinder. The extracted oil collects in the oil drain beneath the press, while the pressed residue (cake) is discharged as pellets through a regulating conical sieve at the outlet.

During each experiment, pressure in the zeer and raw material temperature were recorded. After processing each 5 kg batch, the mass of the extracted oil was measured to determine the oil yield relative to the initial mass.

Grinding efficiency is determined by the degree of pulverization. The physical meaning of the degree of particle size reduction of a material is equal to the ratio of sizes before grinding (D) and after grinding (d), as per the following:

(1) $$i=\frac{D}{d},$$

where D and d are the average particle sizes before and after grinding.

If the particle shape is geometrically incorrect, the geometric mean size is as follows:

(2) $$d=\sqrt[{3}]{l\cdot b\cdot h},$$

where l, b, and h are the greatest length, width, and height of the particle.

The experimental data obtained give a sufficiently complete assessment of the processes that occur during the pressing of the initial mixture of safflower seeds on a single-screw press.

### 2.2. Determination of Pressure and Temperature Changes During the Grinding and Pressing Process

To monitor pressure and temperature changes during the crushing and pressing process of safflower oil, the Xplorer GLX (Roseville, CA, USA, Pasco Scientific company) universal instrument was utilized. The Xplorer GLX is a state-of-the-art device renowned for its precision and versatility. It was employed to measure product temperature and pressure during pressing with high accuracy. The experiments were conducted in triplicate, and the mean values for all parameters were obtained.

By graphically analyzing the recorded data, the required numerical values were determined. The Xplorer GLX features built-in data storage capabilities, allowing it to retain measurement results independently, without the need for a computer. Beyond pressure and temperature, the device can measure numerous additional parameters using its 24 compatible sensors. Key sensors used in this study included the PS-2146 for pressure measurement and the PS-2153 for temperature measurement [33,34]. This advanced setup enabled reliable and precise monitoring of critical parameters during the safflower oil production process.

### 2.3. Determination of Energy Characteristics of the Equipment

During the research on the combined pressing process, the energy characteristics of the experimental pressing equipment were analyzed. The primary objective was to determine the energy expenditure associated with various factors influencing process intensity, including changes in the main geometric dimensions of the press screw mechanism [35]. The energy characterization was conducted using a specialized measuring stand, which is housed in the “Improvement of Technique and Technology of Food Products” laboratory at Shakarim University in Semey.

The measuring device consists of a voltmeter, an ammeter, and a phase meter, which measures the power factor (cos*φ*). These instruments are connected to both the electric motor of the experimental safflower oil press and the electric circuit control system.

The electrical readings are recorded using a web camera, and the power is calculated using the following formula:

(3) $$N=\sqrt{3\cdot U\cdot I\cdot \cos\varphi },$$

where U is the voltage (V), obtained from voltmeter readings, I is the current (A), obtained from ammeter readings, and cos⁡φ is the power factor.

### 2.4. Determination of Compression and Rheological Changes in Safflower Products

The density of safflower cake was measured using hydrostatic scales. For this experiment, three samples of 5 g each were prepared. The samples were initially weighed on an analytical scale and then placed in a stainless-steel mesh basket with a thickness of 0.6–0.8 mm. The basket was secured with a stainless-steel lid.

Prior to the test, the mass of the empty mesh basket with its hangers was measured. The basket containing the sample was then immersed in a measuring beaker filled with ethyl ether. The beaker was placed in a thermostatic vessel maintained at a water temperature of 20 ± 0.5 °C. To ensure the removal of air bubbles from the mesh basket and the sample, the ether in the beaker was stirred thoroughly.

The hydrostatic scales were adjusted using weights hung on the arm of the balance bar, allowing for precise determination of sample density [36].

The volume of safflower cake in each sample, V (m^3^), was calculated using the following formula:

(4) $$V=\frac{m_{w}-m_{s}}{\rho_{l}},$$

where $m_{w}$ is the mass of the weight (kg), $m_{s}$ is the mass of the sample (kg), and $\rho_{l}$ is the density of ether at 20° C (kg/m^3^).

The density of the safflower cake for each sample was determined as follows:

(5) $$\rho =\frac{m_{s}}{V},$$

where $m_{s}$ is the mass of the sample (kg), and V is the volume of the sample (m^3^).

To measure yield stress, the Structurometer ST-2 device for solid and plastic materials was used. This device evaluates the quality of mixtures, semi-finished, and finished products in food production based on both conventional and classical rheological characteristics [37]. The Structurometer ST-2 was developed by the Russian research and production company Radius in compliance with TU 2011-011-17326295-01. The testing process and data analysis were automated using specialized software that controls the structurometer and processes the results.

Before starting the experiment, the device was configured with the appropriate operating mode. For analyzing the yield stress of safflower cake, a cone indenter with a cone angle of 45° was employed.

The test sample was carefully packed into a cylindrical container using a spatula. The container was then placed on the device’s adjustable table to align its height precisely with the instrument. Using the software’s control commands, the table was positioned optimally for measurement.

After pressing the “start” button, the cone indenter automatically descended into the sample, performing the test. The software calculated the instrument’s readings in real time. Once the indenter completed its motion and returned to its original position, the test was concluded by pressing the “stop” button.

Experiments consisting of 5–6 repetitions were performed, and mean values for all indices were obtained. The experimental results, including tables and graphs, were automatically saved for further analysis.

### 2.5. Determination of Physicochemical Parameters of Safflower Oil

The density and specific gravity of safflower oil were measured in accordance with GOST 18848-2019 using the AON-1 hydrometer manufactured by JSC “Steklopribor” [38]. The refractive index was determined using the refractometric method outlined in GOST at a temperature of 20 °C, with the arithmetic mean of three parallel measurements taken as the result.

The density measurement involved immersing the hydrometer into the oil sample, recording the scale reading at the measurement temperature, and converting the results to a density value at 20 °C.

The viscosity of safflower oil was analyzed using a Brookfield DV-E viscometer (manufacturer AMETEK Brookfield, Middleboro, MA, USA), in accordance with GOST 33768-2015 [39], which specifies the method for determining kinematic viscosity and calculating dynamic viscosity of both transparent and opaque liquids. The procedure involved measuring the flow time of a specific oil volume through a glass capillary viscometer under the influence of gravity.

Additional physicochemical properties such as the iodine number, acid number, peroxide number, and saponification number were determined following the methodologies described in AOAC (2005) [40,41].

The residual oil content in safflower cake was determined by the method according to GOST 13979.2-94 [42].

The mass fraction of crude fat and extractive substances in percent was calculated according to the following formula:

(6) $$X_{1}=\frac{(m_{1\;}-m_{2})\cdot 100}{m},$$

where m is the mass of the test product (g), $m_{1}$ is the mass of the flask with oil (g), and $m_{2}$ is the mass of the empty flask (g).

The mass fraction of fat and extractive substances in terms of total dry matter in percent was calculated according to the following formula:

(7) $$X_{1}=\frac{X\cdot 100}{100-W},$$

where X is the mass fraction of crude fat and extractive substances of the tested product at actual moisture content (%), and W is the humidity of the tested product (%).

Experiments consisting of 4 repetitions were performed, and mean values for all indices were obtained.

### 2.6. Determination of Fatty Acid Composition of Safflower Oil

The fatty acid composition of safflower oil was analyzed using a SP-2560 capillary column and a Chromotek 5000.1 gas chromatograph (Closed Joint-Stock Company Special Design Bureau “Chromotek”, Yoshkar-Ola, Republic of Mari El). During chromatographic analysis, one of the most critical and challenging steps was the accurate identification of peaks. The area normalization method was employed to quantify the content of each fatty acid [43].

To prepare the samples, 200 µL of safflower oil was mixed with 2 mL of 2M potassium hydroxide and 4 mL of hexane in methanol to isolate the resulting methyl ester. The solution was then centrifuged at 4000 rpm for 2 min to separate the methanol and hexane layers. A total of 800 µL of hexane and 200 µL of the separated hexane from the upper layer were collected. Alkali was added to the solution to break down triglycerides, while methanol reacted with the fatty acids to form fatty acid methyl esters (FAMEs). The fatty acid composition was then determined using gas chromatography.

To reduce methyl alcohol concentration in the fatty acid solution, 200 µL of separated hexane and 800 µL of pure hexane were added. The experiments were conducted in triplicate, and average values for all parameters were recorded.

### 2.7. Regression Analysis

Multiple regression analysis in this study was conducted using the ordinary least squares (OLS) technique, a widely used method for estimating linear relationships between independent and dependent variables. The primary objective of applying OLS was to evaluate the effects of pressing time, degree of grinding, pressure, and temperature on safflower oil yield.

Before conducting the analysis, experimental data were carefully prepared to ensure that each variable was adequately represented. The dataset was structured in an organized manner, with the dependent variable (oil yield) clearly separated from the independent variables. Additionally, an intercept term (constant) was included in the regression model to account for the baseline oil yield when all independent variables are zero.

To ensure the validity of the OLS regression, key statistical assumptions were verified, including linearity, independence of errors, homoscedasticity (constant variance of errors), normality of residuals, and absence of multicollinearity among independent variables. The estimation was performed using Python 3.13 and the statsmodels package, allowing for precise calculation of coefficients, standard errors, *t*-statistics, and *p*-values for all predictors.

The model’s goodness of fit was assessed using *R*-squared and adjusted *R*-squared metrics, which indicate the proportion of variance in oil yield explained by the independent variables. To further validate the model, significance tests were conducted, including *t*-tests for individual predictors and an *F*-test for overall model significance.

Additionally, a sensitivity analysis was performed to examine how small variations in independent variables impact the projected oil yield, further strengthening the model’s reliability for practical applications. These rigorous statistical checks confirm the robustness of the regression model, making it a valuable tool for optimizing safflower oil extraction processes.

## 3. Results and Discussion

### 3.1. Study of Safflower Oil Extraction at Different Pressing Times and Degrees of Grinding

To evaluate the quantitative and qualitative parameters of safflower oil extraction, experiments were conducted under the following conditions:

- Pressing Times: τ=3.41 s, τ=4.13 s, τ=5.27 s, τ=6.23 s;
- Degrees of Grinding: i=0.125, i=0.25, i=0.5, i=1.

The parameter “pressing time” was evaluated as an important factor affecting the pressing efficiency. Excessive pressing time could reduce the oil yield, and a short pressing time could lead to high oil content in the cake. The total pressing time was determined using the following relationship based on time and screw speed. The formula was used to calculate the pressing time at a known rotational speed:

(8) $$t=\frac{L}{v},$$

where t is the pressing time (min), L is the press chamber length (m), and v is the feed rate of raw material (m/s), which depends on screw speed.

**Figure 6:**
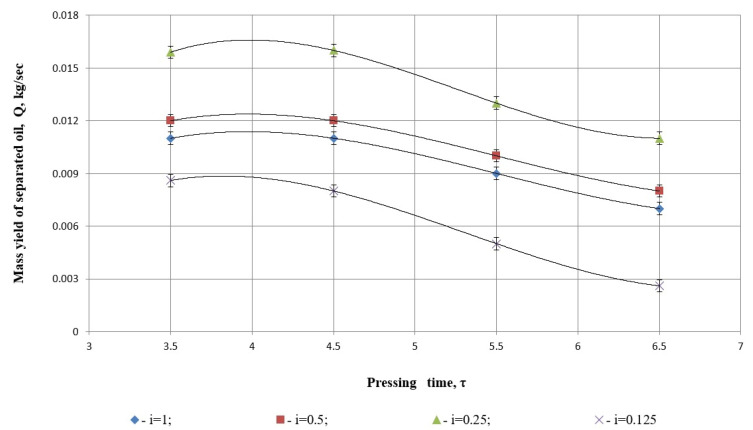
Dependence of mass yield of separated oil on pressing time and different degrees of grinding.

As shown in Figure 6, analysis of the oil yield at various degrees of grinding revealed that the optimal conditions for the pressing process are a pressing time of τ=3.41 s and a grinding degree of i=0.25.

At lower screw rotation speeds, the reduced throughput limits the oil extraction due to the material balance constraints, resulting in decreased oil yield. Conversely, excessively high rotation speeds reduce pressing time, leading to insufficient separation of oil from safflower seeds and a significant increase in energy consumption. Consequently, reducing the pressing time below τ=3.41 s does not yield further improvements.

When we examine the graph in Figure 6, the relationship between degrees of grinding and oil yield becomes evident. Smaller particle sizes require shorter pressing times, but degrees of grinding beyond i=0.25 lead to a sharp decline in oil yield. This decrease is particularly pronounced at i=0.125 and i=1, which can be attributed to changes in the viscosity of the material. Excessive grinding at these levels closes the natural filter channels within the product matrix, hindering effective oil separation.

Quadratic regression analysis of Figure 6 illustrates the relationship between pressing time *τ* and the mass yield of separated oil *Q* (in kg/s), for different values of the parameter *i*. For each case, a second-degree polynomial of the form (Table 1) was fitted to the data:

(9) $$Q(\tau )={a\tau }^{2}+b\tau +c,$$

#### 3.1.1. Polynomial Coefficients

- For all values of the parameter *i*, the coefficient *a* is negative. This indicates a concave-down parabola, meaning that each function has a distinct maximum, an optimal pressing time beyond which the oil yield starts to decrease;
- The coefficient *b* is positive in each case, which reflects an initial increase in oil yield as pressing time increases.

#### 3.1.2. Model Fit Quality (*R*^2^ Values):

- The coefficient of determination *R*^2^ is high across all models, suggesting an excellent fit to the data:
  *R*^2^ = 0.995 for *i* = 0.125—the best fit;
  *R*^2^ = 0.987 for *i* = 0.5.

Slightly lower *R*^2^ values were observed for *i* = 1 (0.91) and *i* = 0.25 (0.88).

#### 3.1.3. Interpretation of Parameter *i*

- The highest yield was achieved at *i* = 0.25, which aligns with the peak visually observable in the plot, indicating the most efficient pressing conditions;
- Both overly high (*i* = 1) and overly low (*i* = 0.125) values of the parameter resulted in decreased yields, suggesting the existence of an optimal operating condition.

Quadratic regression analysis reveals a linear–nonlinear relationship between pressing time and separated oil mass yield that graphs as a concave-down parabola for all conditions tested. The negative value of the quadratic coefficients (a) at all levels of process parameter iii indicates that the oil yield becomes better with pressing time up to a certain point, when it begins to decrease. Such a trend suggests that there might be an optimal period for pressing for efficiency in extraction. Such large *R*^2^ values bear witness to the efficiency of models used here, characterizing well the observed tendencies and proving their predictiveness and suitability for optimization applications.

Mizera et al. (2018) [14] investigated the pressing of rapeseed using a Farmer 20 Duo screw press (Farmer 20, Farmet AS, Ceska Skalice, Czech Republic) at screw speeds of 10, 20, 30, 40, 55, and 65 rpm. Their findings showed that the maximum oil extraction efficiency of 82.6% occurred at the lowest screw speed and longest pressing time. However, it is important to note that their study involved a different seed type and equipment specification compared with the present research.

Similarly, the results align with the findings of Bogaert et al. (2018) [11], which indicate that increasing screw speed can reduce both oil yield and specific energy consumption. This highlights the necessity of optimizing screw speed to balance extraction efficiency and energy use.

It was determined that the optimal pressing conditions are achieved at a pressing time of τ=3.41 s and a grinding degree of i=0.25.

**Figure 7:**
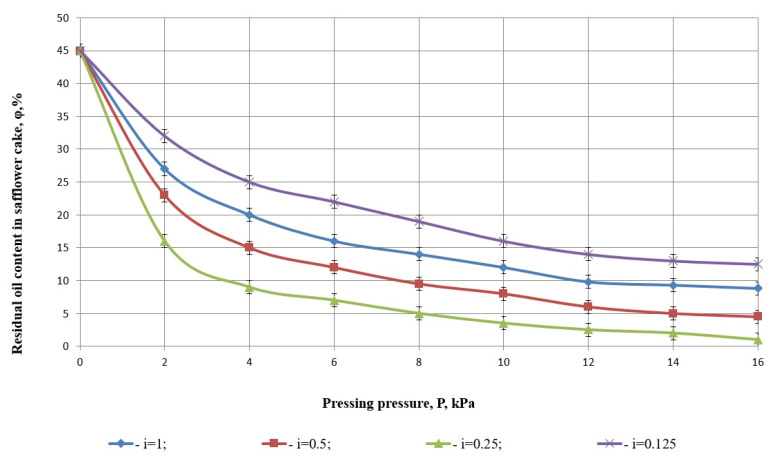
Dependence of residual fat content of safflower cake on pressure and different degrees of grinding.

Pressure is one of the key parameters influencing the efficiency of the pressing process [43]. Analyzing the graph in Figure 7, which illustrates the relationship between grinding degree and pressure during pressing, reveals important insights.

Residual oil content is an important indicator of the efficiency of the pressing process and the quality of the resulting oil.

At a degree of grinding of i=0.25 and a pressure of P=8 kPa, the residual fat content in the cake reaches its minimum level, indicating optimal oil extraction. Beyond this pressure, no significant reduction in residual fat is observed, suggesting that further increases in pressure during pressing are ineffective.

For other degrees of grinding, the observed dependence of residual fat content on pressure during pressing can be attributed to the closure of the product’s natural filter channels. Excessive grinding creates finer particles that impede oil flow, reducing the efficiency of the pressing process. It is known that a low residual oil content indicates a high efficiency of the extracted oil because most of the oil has been removed from the raw material. In contrast, high residual oil content may indicate an inefficient pressing process or equipment problems [44,45].

Figure 7 illustrates the relationship between pressing pressure (*P*, in kPa) and the residual oil content in safflower cake (%, *y*-axis) for different degrees of grinding (*i*), as follows (Table 2):

- i=1—whole seeds (blue diamonds);
- i=0.5—moderately ground (red squares);
- i=0.25—optimally ground (green triangles);
- i=0.125—very finely ground (purple Xs).

Each curve represents a third-degree polynomial regression (cubic function), and the respective regression equations and coefficients of determination *R*^2^ are shown on the graph.

- *i* = 0.25 (green triangles), *R*^2^ = 0.9776:

(10) $$y=-0.0894x^{3}+0.9557x^{2}-10.102x+43.051$$

This model has the highest coefficient of determination, explaining 97.76% of the variability in residual oil content. This is the best-fitting model, both statistically and technologically, indicating optimal pressing conditions.

- *i* = 0.125 (purple Xs), *R*^2^ = 0.9678:

(11) $$y=-0.0252x^{3}+0.8219x^{2}-8.896x+42.87$$

This is also a very good model fit. It explains 96.78% of the variability. However, the residual oil content remains higher than at *i* = 0.25, likely owing to over-grinding, which clogs natural filtration channels and impairs oil flow.

- *i* = 0.5 (red squares), *R*^2^ = 0.9574:

(12) $$y=-0.0179x^{3}+0.3666x^{2}-3.7347x+42.036$$

This is also a very good fit that explains 95.74% of the variance in the residual oil content. It is slightly worse than the one for *i* = 0.25, but it is still reliable for practical use.

- *i* = 1.0 (blue diamonds), *R*^2^ = 0.9504:

(13) $$y=-0.0059x^{3}+0.1283x^{2}-1.3934x+43.162$$

This model explains 95.04% of the variation in the data. Though still statistically valid, it is the least accurate among the four and corresponds to unprocessed whole seeds, which provide the lowest oil extraction efficiency.

Statistical treatment of the regression equations unambiguously indicates that the relationship between pressing pressure and residual oil content in safflower cake is not linear and hence presumably can be most adequately described by cubic polynomial functions. The degree of grinding *i* = 0.25 model of all examined ones not only had the maximum value of the coefficient of determination (*R*^2^ = 0.9776), but also the optimal practical result—minimum residual oil content. This implies that there is an optimum degree of grinding that creates optimal structural conditions within the seed matrix for efficient pressure-release oil drainage. Under-grinding (*i* = 1) and over-grinding (*i* = 0.125) yielded worse regression fits and higher residual oil content, lending support to the contention that excessive particle sizes hinder efficient oil separation, either by too little mechanical rupture or clogging of natural filtration channels.

The experimental study concludes that intensification of the pressing process cannot be achieved solely by increasing pressure. Instead, it is necessary to experimentally determine an optimal pressure value, considering the technological characteristics of production, the structural and mechanical properties of the material, and the quantity and quality of the extracted liquid.

### 3.2. Determination of Power Requirements for the Pressing Process at Different Degrees of Grinding and Pressing Times

The pressing process is known to be one of the most energy-intensive operations. Combining grinding and pressing processes, while quantifying the power required for both, adds a quantitative dimension to this research and provides insights into process optimization.

**Figure 8:**
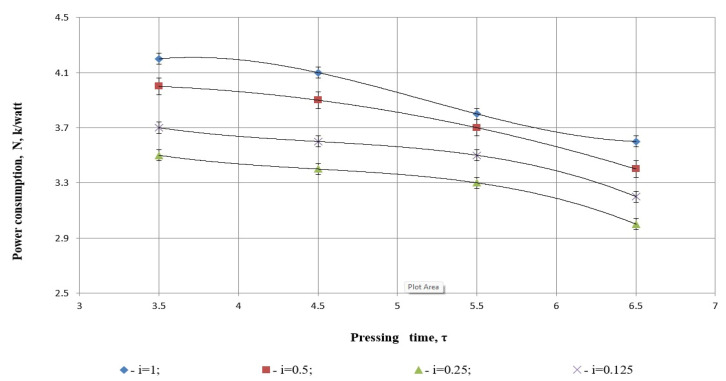
Power dependence on pressing time and different degrees of grinding.

Figure 8 shows the relationship between pressing time (*τ*) and energy consumption (Capacity, N·kWatt) at various degrees of grinding (*i*). Each curve represents a cubic polynomial regression fitted to the data, with corresponding equations and coefficients of determination *R*^2^ provided on the plot (Table 3).

Each degree of grinding *i* has its own fitted cubic polynomial. The statistical measure of interest is the coefficient of determination (*R*^2^), which indicates how well the model explains the variability in the observed data.

- *i* = 1 (blue diamonds), *R*^2^ = 1:

(14) $$y=0.05x^{3}-0.775x^{2}+3.6875x-1.3563$$

The regression model for *i* = 1 perfectly fits the observed data, as shown by the coefficient *R*^2^ = 1. This means the model explains 100% of the variability in energy consumption based on pressing time. This result strongly indicates a deterministic, well-behaved trend in the data, likely due to high experimental consistency at this degree of grinding.

- *i* = 0.5 (red squares), *R*^2^ = 1:

(15) $$y=10^{-14}x^{3}-0.055x^{2}+0.3x-3.5625$$

Again, we observe a perfect statistical fit for this model. The cubic term is effectively zero, indicating that a quadratic function might be sufficient to describe this trend. However, the *R*^2^ value of 1 confirms that the regression surface passes through all data points with no deviation.

- *i* = 0.25 (green triangles), *R*^2^ = 1:

(16) $$y=-0.0333x^{3}+0.45x^{2}-2.0917x+6.7375$$

This degree of grinding also yields a model with perfect goodness-of-fit. The shape of the curve indicates a downward trend in energy consumption with increasing pressing time, which may reflect improved flow dynamics or material plasticity during prolonged pressing.

- *i* = 0.125 (purple Xs), *R*^2^ = 1:

(17) $$y=-0.0333x^{3}+0.45x^{2}-2.0917x+6.9375$$

This model has an identical structure to the one for *i* = 0.25, but a slightly higher constant term. Again, the *R*^2^ = 1 indicates that the model fits the data exactly, implying highly regular behavior in energy consumption under this grinding condition.

The statistical evaluation of Figure 8 confirms that the dependency between pressing time and energy demand (capacity) is best described by the applied cubic regression models used, and all degrees of grinding (*i* = 1.0, 0.5, 0.25, and 0.125) possess a coefficient of determination *R*^2^ = 1. This implies that the models describe the experimental data precisely, without remaining variance in the observed interval. This level of exact fit is characteristic of very uniform experimental and data-gathering conditions. It also means, however, that models are assumed to interpolate in a small set of data points, so the models are theoretically precise but possibly sensitive to deviations from the sampled values. Every one of the regression lines’ typical shapes reflects the way particle grinding level affects the material’s mechanical resistivity, in which fine particles will lead to low energy requirements for long pressing times.

As illustrated in Figure 8, the relationship between power consumption, pressing time, and degree of grinding was analyzed. The results indicate that power consumption increases as the pressing time is reduced. This is attributed to the intensified oil separation process during shorter pressing intervals, where increased oil extraction correlates with higher energy usage. For example, when the time taken varied from 3.5 to 6.5 s, the power consumption varied with the degree of grinding and time, from 3.6 kWt without pre-crushing to 4.2 kWt when the seeds were crushed to 2 mm.

The study found that at a degree of grinding of i=0.25, power consumption was at its lowest level. Conversely, the maximum power consumption occurred at i=1, where the pressing process was carried out without any pre-milling. This increase in power demand is explained by the higher oil separation pressure and greater internal and external friction in the unprocessed product.

At a degree of grinding of i=0.125, the higher power requirement is attributed to the energy-intensive nature of the grinding process itself, which outweighs the energy demands of the pressing process. Therefore, i=0.25 emerges as the most efficient grinding parameter, balancing energy consumption and oil separation performance. The findings of this study closely align with those reported by Kabulov et al. (2012), which demonstrated that an increase in screw rotation speed during sunflower seed pressing leads to a corresponding rise in power consumption [46]. Similarly, Korendiy et al. developed a novel press featuring two compression stages for vegetable oil extraction. Their experiments revealed that this design achieves an oil yield exceeding 90%, outperforming traditional presses. The final phase of their research focused on the energy characterization of the developed equipment, particularly analyzing power consumption during oil extraction and cake discharge [47].

As a result, it was determined that further reduction of the safflower seed degree of grinding below i=0.25 does not yield beneficial results. Instead, it leads to a decline in pressing efficiency and an increase in power consumption.

### 3.3. Compression and Rheological Changes of Safflower Product During Pressing at Different Degrees of Grinding

Among the physical properties of safflower cake during the pressing process, density plays a pivotal role. The density of the cake serves as a key parameter in characterizing the degree of compression during the pressing process.

**Figure 9:**
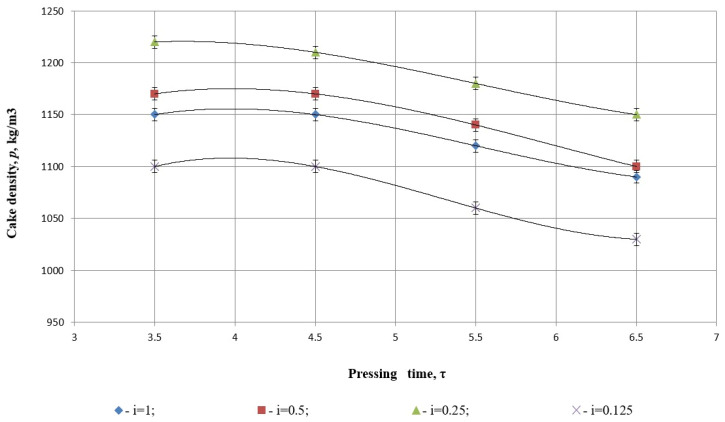
Dependence of cake density on pressing time and different degrees of grinding.

The variation in cake density as a function of pressing time and degree of grinding was analyzed (as seen in Figure 9). The findings revealed that under the influence of external forces during pressing, the density of safflower cake increases, irrespective of the liquid fraction remaining in the cake. However, upon the cessation of external forces, the density decreases due to the cake’s inherent elasticity, which is influenced by the liquid fraction retained within the cake.

These observations highlight the interplay between external compressive forces and the rheological properties of the product, emphasizing the importance of optimizing grinding and pressing parameters to achieve the desired product quality and minimize energy losses.

Figure 9 illustrates the relationship between pressing time (τ) and cake density (ρ, kg/m^3^) for various degrees of grinding, denoted as *i* = 1, 0.5, 0.25, 0.125. Each dataset is modeled using a cubic polynomial regression, and the goodness-of-fit of each model is evaluated using the coefficient of determination *R*^2^ (Table 4).

The cake density curves are fitted with third-degree polynomials. Each regression equation is explicitly shown on the graph, along with its corresponding *R*^2^ value. All models report *R*^2^ = 1, suggesting a perfect fit to the experimental data.

- *i* = 0.25 (green triangles), *R*^2^ = 1:

(18) $$y=5x^{3}-82.5x^{2}+418.75x+480.62$$

The regression model shows a perfect fit, indicating that the cubic polynomial explains 100% of the variance in cake density across the measured pressing times. The high-order terms suggest a non-linear dynamic, and the curve shape indicates a density peak at moderate pressing times, followed by a slight decline. This likely reflects compaction followed by material relaxation or internal resistance build-up.

- *i* = 0.5 (red squares), *R*^2^ = 1:

(19) $$y=3.333x^{3}-60x^{2}+319.17x+645$$

Another case of the perfect fit, with slightly lower initial density values than the *i* = 0.25 case. The regression terms describe a similar curvature trend, suggesting that density initially increases or stabilizes and then decreases slightly with longer pressing durations. The perfect *R*^2^ implies no deviation between observed and predicted data points.

- *i* = 1.0 (blue diamonds), *R*^2^ = 1:

(20) $$y=3.333x^{3}-55x^{2}+269.17x+808.75$$

Despite the relatively coarse material (*i* = 1), the data still fit the cubic model exactly. The intercept and coefficients reflect higher initial density compared to more finely ground material, possibly due to larger particles resisting compaction. The curve shows a slower density decline over time, which may indicate a different internal structure evolution under pressure.

- *i* = 0.125 (purple Xs), *R*^2^ = 1:

(21) $$y=-8.333x^{3}+132.5x^{2}-657.92x+63.125$$

Although this model also exhibits a perfect statistical fit, the negative cubic term and strong curvature suggest a steeper and more irregular response. The finely ground material seems to reach a density peak early, then undergoes a sharper decline. This may point to structural instability or the collapse of natural oil-flow channels due to excessive pulverization.

Statistical analysis of Figure 9 shows a highly coherent and accurate cubic regression model fit to the experimental data, where all four levels of grind (*i* = 1, 0.5, 0.25, and 0.125) yielded the coefficient of determination *R*^2^ = 1. This perfect statistical fit indicates that the polynomial equations account for all the variation in cake density with pressing time across the data range being measured. Of most interest, the models suggest various compaction behaviors depending on the degree of grinding: a moderate grinding (*i* = 0.25 and 0.5) exhibits higher initial cake density and a gradual decline with time, while over-grinding (*i* = 0.125) produces a sharp drop, the likely reason being the collapse of the structural matrix. Even with the ideal fit, the consistently high *R*^2^ values mean that the models are interpolative and can, in theory, overfit to sparse data points, so extrapolation beyond experimental conditions should be performed with caution.

According to Figure 9, the density of safflower cake decreases during the pressing experiment owing to the reduction of fat content within the cake. On the contrary, the modulus of elasticity increases because oil removal takes place during safflower pressing, which leads to a decrease in cake density due to a decrease in mass and pore formation. However, at the same time, the elastic modulus increases because the structure becomes more cohesive and stable, which is given in the work by Paronyan [48].

**Figure 10:**
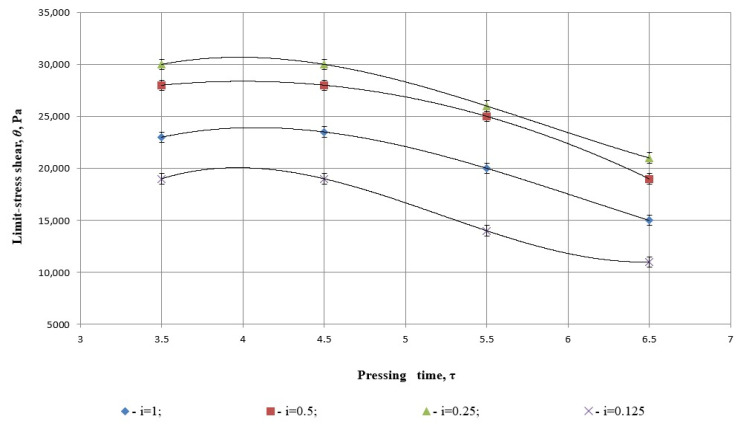
Dependence of the yield stress of the cake on pressing time and different degrees of grinding.

The study of the structural and mechanical properties of safflower cake during pressing highlights the importance of yield stress. As shown in Figure 10, the maximum yield stress was observed at a degree of grinding of i=0.25 and a pressing time of τ=3.41 s. This change can be attributed to the low residual fat content in the cake under these conditions, reflecting the optimal balance between grinding and pressing parameters.

It is important to note that, in addition to the liquid fraction, yield stress values are potentially influenced by temperature differences. However, the experimental results did not establish a significant correlation between temperature variation and yield stress, as indicated in the referenced figure.

Figure 10 illustrates the relationship between pressing time (*τ*) and limit shear stress (*θ*, Pa) of safflower press cake under varying degrees of grinding *i*. Each dataset is modeled using a third-degree polynomial regression, and the strength of fit is evaluated using the coefficient of determination *R*^2^ (Table 5).

All models presented in Figure 10 report a coefficient of determination *R*^2^ = 1.

This means that each regression curve fits the corresponding data perfectly, with 100% of the variation in shear stress explained by the model. While mathematically ideal, this may reflect interpolation over a small dataset and should be interpreted with care in predictive applications.

- *i* = 0.25 (green triangles), *R*^2^ = 1:

(22) $$y=-1500x^{3}+11200x^{2}-41300x+46314$$

This model represents the highest limit shear stress across all pressing times. The curve has a concave shape, peaking at moderate pressing durations and slightly decreasing thereafter. The perfect *R*^2^ confirms a precise mathematical fit, and the behavior likely corresponds to an optimally compacted cake structure that weakens only slightly over time.

- *i* = 0.5 (red squares), *R*^2^ = 1:

(23) $$y=500x^{3}-8750x^{2}+45875x-44813$$

This curve is relatively flat, indicating that the shear stress remains mechanically stable over time at this degree of grinding. The perfect fit suggests high consistency in material behavior. The peak value is slightly lower than for *i* = 0.25, which aligns with slightly reduced structural compactness.

- *i* = 1.0 (blue diamonds), *R*^2^ = 1:

(24) $$y=116.67x^{3}-3625x^{2}+41390x-46314$$

This model reflects lower shear strength, likely due to insufficient seed breakdown in whole (unmilled) material. The polynomial shows a more pronounced decline in stress over time, likely due to internal voids and low cohesion. Despite this, the model still captures the trend with complete precision.

- *i* = 0.125 (purple Xs), *R*^2^ = 1:

(25) $$y=-116.67x^{3}+18250x^{2}-89708x-121438$$

Although the statistical fit is again perfect, this curve exhibits the most dramatic decline in shear stress as pressing time increases. This strongly suggests that over-grinding compromises the structural integrity of the press cake. The initial high stress may result from early compaction, followed by weakening due to collapse of microchannels or excessive fragmentation.

The statistical analysis of Figure 10 reveals that the cubic polynomial regressions used to model the relationship between pressing time and the limit shear stress of safflower press cake exhibit a perfect fit across all degrees of grinding, with each model achieving a coefficient of determination *R*^2^ = 1. This indicates that the mathematical models fully capture the variability in the data, reflecting highly consistent experimental conditions. Among the variants, the shear stress reaches its maximum values for moderately ground material (*i* = 0.25), suggesting optimal structural cohesion, while both over-ground (*i* = 0.125) and unground seeds (*i* = 1) display significantly lower and less stable mechanical resistance over time. The steep decline in shear stress for the finest degree of grinding further suggests a weakening of the internal cake structure due to excessive particle breakdown, confirming that moderate grinding provides the most mechanically resilient pressing conditions.

As illustrated in Figure 10, the yield stress (*τ*) is influenced by both the fat content and the temperature of the safflower cake. However, as oil is separated during the pressing process, the dependence of yield stress on cake temperature diminishes.

In the experimental study, safflower grains underwent heat treatment within an effective temperature range of t=353–358 K, factoring in the additional temperature increase caused by internal friction within the working components during the pressing process.

**Figure 11:**
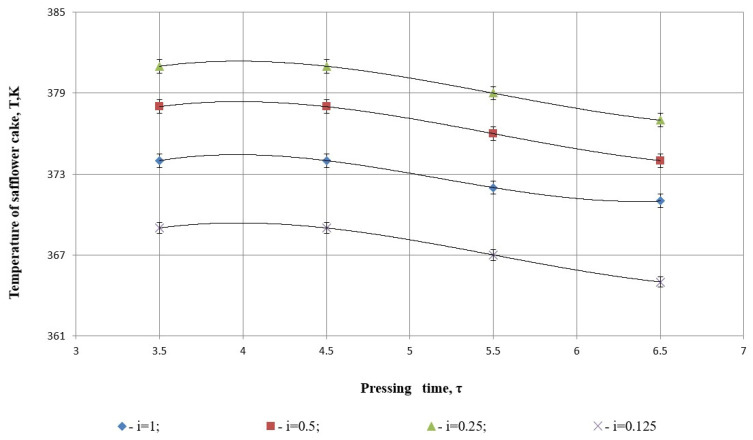
The dependence of cake temperature on pressing time and different degrees of grinding.

Figure 11 highlights that variations in cake temperature during pressing are primarily influenced by the separation of the liquid fraction and the pressing time. A lower rotation speed of the pressing screw intensifies the temperature’s effect on the liquid fraction separation while reducing its dependence on pressing time.

Figure 11 presents the relationship between pressing time (*τ*, in minutes) and the temperature of safflower cake (*T*, in Kelvin) across different degrees of grinding, *i* = 1, 0.5, 0.25, 0.125. As in the previous figures, each dataset is fitted using a cubic polynomial regression, and the fit quality is assessed by the coefficient of determination *R*^2^, which equals 1.000 for all cases (Table 6).

All models exhibit a coefficient of determination *R*^2^ = 1, indicating a perfect statistical fit to the measured data points. This suggests that the polynomial regressions explain 100% of the variability in temperature for each degree of grinding, with no residual error.

- *i* = 0.25 (green triangles), *R*^2^ = 1:

(26) $$y=0.5x^{3}-9.833x^{2}+27.9178x+333.3$$

The temperature curve shows a slight peak and then a gradual decrease with longer pressing times. The low magnitude of coefficients indicates mild nonlinearity. The perfect fit means this model passes precisely through all observed data points and captures the nuanced thermal response of moderately ground material.

- *i* = 0.5 (red squares), *R*^2^ = 1:

(27) $$y=0.3333x^{3}-5.5x^{2}+27.917x+336.37$$

This model also reflects a small thermal curvature, with slightly lower maximum temperature than for *i* = 0.25. The curve is nearly parallel in shape, suggesting similar thermal dynamics but at a marginally reduced energy dissipation. Again, the *R*^2^ = 1 confirms perfect mathematical alignment.

- *i* = 1 (blue diamonds), *R*^2^ = 1:

(28) $$y=0.3333x^{3}-5.5x^{2}+27.917x+333.3$$

Structurally identical to the model for *i* = 0.5, but with a lower intercept. The regression perfectly captures the more modest rise and decline in temperature for whole seeds, possibly due to lower heat generation from reduced internal friction or compaction resistance.

- *i* = 0.125 (purple Xs), *R*^2^ = 1:

(29) $$y=0.3333x^{3}-5.5x^{2}+27.917x+324.37$$

The negative sign on the cubic and quadratic terms indicates a mirror curvature compared to other curves. This model suggests a steeper and more linear-like temperature decrease with pressing time, likely due to over-grinding, which diminishes internal structural resistance and reduces internal heat buildup. Despite this behavioral deviation, the model maintains perfect fidelity to the experimental data.

Statistical fit of Figure 11 is in line with maintaining that cubic re-gradients models are best at defining the press time vs. safflower cake temperature relation for each degree of grinding on the premise of assuming a determination coefficient *R*^2^ = 1 in each case. The ideal fit guarantees that models account for all variations of temperature across the range of measurement, which is characteristic of very replicable experimental data. The trends with the measurements indicate that moderately ground material (*i* = 0.25 and 0.5) is warmest, with a consistent decrease as pressing advances, perhaps due to the balance between thermal dissipation and internal friction. Highly ground material (*i* = 0.125), on the other hand, indicates a consistent decrease in temperature, which suggests that over-grinding reduces internal resistance and inhibits heat formation during pressing. These trends, well represented by the regressions, show that the degree of grinding determines the thermal response of the pressed material.

As shown in Figure 11, the experimental results reveal that changes in both pressure and power are influenced by the same set of factors.

These findings align closely with the study by Abdilova et al. (2023) [17], which examined temperature variations during the pressing of sunflower seeds at different screw speeds. At a screw speed of 30 rpm, the cake temperature ranged from 93 to 106 °C. Increasing the speed to 120 rpm resulted in temperatures between 95 and 107 °C, while at 250 rpm, the temperature reached 108 °C.

### 3.4. Physicochemical Parameters of Safflower Oil

The quality of safflower oil is largely determined by its physicochemical characteristics. The experiments conducted using a conventional screw press were carried out at process parameters similar to those of the experimental press combining the grinding and pressing processes, which yielded the results summarized in Table 7. These findings provide a comparative evaluation of the oil produced by the two methods, highlighting the impact of the combined process on product quality. To ensure the reliability of the results and to exclude the influence of differences in raw materials, safflower seeds from the same batch were used in all series of experiments.

The results presented in Table 7 demonstrate notable differences in the physicochemical properties of safflower oil extracted using the existing screw press compared with the modernized screw press.

- Density: The density of safflower oil after pressing with the existing screw press was 0.922 g/mL, whereas a slight decrease to 0.917 g/mL was observed with the modernized press;
- Specific Gravity: The specific gravity increased from 0.927 to 0.960, likely due to the higher proportion of polyunsaturated fatty acids obtained with the modernized press;
- Refractive Index: The refractive index also showed an increase, from 1.443 with the existing press to 1.464 with the modernized version;
- Viscosity: A minor increase in viscosity was observed, from 45.4 cP with the existing press to 45.9 cP with the modernized press;
- Acid Number: The acid number rose slightly, from 1.05 mg KOH/g with the existing press to 1.063 mg KOH/g with the modernized press;
- Iodine Number: The iodine number, an indicator of unsaturation in fatty acids, increased from 144.19 to 147.15 g in I_2_/100 g, confirming a higher proportion of unsaturated fatty acids in the oil extracted using the modernized press;
- Peroxide Number: A reduction in the peroxide number, from 8.04 mol/kg with the existing press to 7.61 mol/kg with the modernized press, suggests improved oxidative stability and enhanced storability;
- Saponification Number: The saponification number increased from 159.3 mg KOH/g with the existing press to 161.6 mg KOH/g with the modernized press, indicating a slightly higher amount of ester bonds in the oil.

These results underscore the improvements achieved by the modernized press in extracting safflower oil with enhanced physicochemical qualities. The observed changes confirm the suitability of this oil for direct consumption as well as for manufacturing oil-based products such as mayonnaise, sauces, and spreads.

Physicochemical variation between the experimental and the traditional screw pressing safflower oil shows similarity and non-detectable variations (Table 8). Most of the parameters, like density, specific weight, refractive index, viscosity, and acid number, were not different (*p* > 0.05), and thus, it implies that the overall structure and rheological behavior of the oils do not vary regardless of the press used. This indicates that the new experimental pressing procedure keeps the overall physical and integrity characteristics of the oil equivalent to those kept by conventional methods. The oil’s density, for example, was essentially the same (0.922 ± 0.190 g/mL vs. 0.917 ± 0.193 g/mL), as well as the viscosity (45.4 ± 1.35 cP vs. 45.9 ± 1.395 cP), with similar thickness and flowing characteristics.

The results also reveal statistically significant enhancement of certain chemical quality parameters of the oil produced using the experimental press. Specifically, the peroxide number was significantly lower (7.61 ± 0.55 mol/kg vs. 8.04 ± 0.54 mol/kg, *p* = 0.043), indicating a lower initial amount of oxidation products and, consequently, the potential for longer shelf life and higher oxidative stability of the experimental oil. In addition, saponification number was significantly higher in the test sample (161.6 ± 2.59 mg KOH/g vs. 159.3 ± 2.54 mg KOH/g, *p* = 0.02) by virtue of either increased amount of short-chain fatty acids or structural integrity of the glyceride molecule. The iodine number was marginally short of being statistically significant (*p* = 0.052), with the corresponding value indicating the marginally raised level of unsaturation in the test oil. These findings verify that the new pressing technology in the experiment group not only maintains the normal physicochemical features but also can enhance the oxidative and composition quality of safflower oil.

### 3.5. Fatty Acid Composition of Safflower Oil

The fatty acid composition is a key determinant of the quality and nutritional value of safflower oil. Table 9 presents a comparative analysis of the fatty acid composition of safflower oil extracted using the existing screw press and the experimental press, which integrates grinding and pressing into a single process. This comparison highlights the impact of the modernized extraction method on the fatty acid profile of the oil.

The outcomes in Table 9 and Table 10 demonstrate that processing using the experimental screw press leads to a stunning surge in the nutritional quality of safflower oil, particularly the content of fatty acids. Most strikingly of all, the level of α-linolenic acid (C_18:3_), an extremely valued omega-3 fatty acid, surges from 0.1 ± 0.062% in the control press to 1.3 ± 0.225% with the experimental process. This is more than a tenfold increase and may be due to improved extraction conditions that favor or preserve the liberation of more susceptible, unsaturated fatty acids. Additionally, oleic acid (C_18:1_) with its positive cardiovascular effects, is raised from 6.3 ± 0.506% to 7.9 ± 0.568%. All these observations show that experimental technology not only preserves but also enhances the functional and health-imparting components of safflower oil.

It is evident in the broader and more varied composition of the identified unsaturated fatty acids of the oil from the experimental screw press. Eicosenoic acids (Ʃ C_20:1_) and erucic acids (Ʃ C_22:1_) content, though low, reflect a rise in the experimental change, from 0.2 ± 0.089% to 0.25 ± 0.101% and from 1.5 ± 0.256% to 2.7 ± 0.327%, respectively. These findings indicate that the experimental press is capable of inflicting less thermal or oxidative stress on the oil matrix and hence recover or extract fewer exotic long-chain fatty acids better. Interestingly, while the comparatively higher total unsaturated acid yields, saturation of fatty acids such as palmitic acid (C_16:0_) and arachidic acid (C_20:0_) also show marginal increases, perhaps due to the overall higher recovery of the oil. Overall, this is a step towards the betterment of the technology that can facilitate more capture of the indigenous lipid profile native to safflower seeds.

The analysis of the fatty acid composition of safflower oil reveals notable changes in various components after processing with the modernized screw press. Among the saturated fatty acids, C_18:0_ (stearic acid) was present at 2.1% in the safflower oil processed by the existing screw press, which decreased slightly to 2% after processing with the modernized press.

An increase in the content of several fatty acids was observed, including C16:1 (palmitoleic acid) to 5%, C_20:0_ (arachidic acid) to 0.9%, C_18:1_ (oleic acid) to 7.9%, C_22:0_ (behenic acid) to 0.28%, Ʃ C_20:1_ (eicosenoic acids) to 0.25%, and Ʃ C_22:1_ (erucic acids) to 2.7%. Notably, the unsaturated fatty acids C_18:2_ (linoleic acid) and C_18:3_ (α-linolenic acid) increased to 85.3% and 1.3%, respectively.

The obtained data align closely with existing literature [49,50], which underscores the reliability and accuracy of the conducted studies. These results further confirm the ability of the modernized press to enhance the unsaturated fatty acid profile of safflower oil, which in turn contributes to its nutritional and functional properties.

Unlike the studies by Kakimov et al. (2024) [51], which focused primarily on optimizing individual aspects of press equipment, or the work of Iskakov et al. (2023) [4], which examined the effects of centrifuge design and parameters on safflower oil quality, this research introduces a novel integration of grinding and pressing processes. By combining these two stages within a single piece of equipment, this approach enhances production efficiency, reduces equipment and operational costs, and minimizes processing time.

This paper proposes a resource-efficient technology tailored for small and medium-sized enterprises, where financial and production resources are often limited, focusing on the mathematical modeling of safflower seed pressing and the development of compact production lines. This work presents an innovative, integrated solution.

Comparative studies were conducted to evaluate the physicochemical and fatty acid composition of safflower oil produced using traditional pressing methods versus the newly developed combined-process equipment. The determination of optimal parameters (e.g., pressing time *τ* = 3.41 and degree of grinding *i* = 0.25) not only enhances technological efficiency but also improves oil quality by increasing fatty acid content. The results indicate that oil obtained using the developed equipment exhibits an improved fatty acid profile, enhancing its biological value and expanding its potential applications beyond direct consumption to include use in oil-based products. Overall, this study marks a significant advancement in safflower oil production technology. It offers a comprehensive, resource-saving approach that can be readily adopted by small and medium-sized enterprises, enhancing both the competitiveness and quality of the final product.

## 4. Conclusions

To enhance the efficiency of the pressing process, an experimental press incorporating a combined grinding mechanism was designed and developed.

The intensification of the pressing process through this combined approach was evaluated using various design parameters and performance metrics. As a result, optimal parameters were identified: a pressing time of τ=3.41 s and a degree of grinding of i=0.25. These conditions delivered the best results in terms of oil yield, residual oil content in safflower cake, press power consumption, rheological properties, and cake temperature.

A comparative analysis of qualitative and quantitative indicators at different degrees of grinding validated the optimal parameters for the combined grinding and pressing processes. A comprehensive evaluation of the results revealed that the processed products exhibited improved consumer properties, along with an increase in the oil’s energy and biological value.

These insights suggest that safflower oil manufacturers should prioritize temperature control and moderate pressure application, along with an optimized degree of grinding, to achieve maximum efficiency. Future research could further explore interactions between these variables, enabling the development of more advanced process control strategies that enhance both the sustainability and economic viability of safflower oil production.

Based on the findings presented in the article, this conclusion focuses specifically on the results of the statistical analyses, which provided compelling evidence for the effectiveness of the innovative approach to safflower processing through the integration of grinding and pressing.

The first most critical outcome of the regression analysis was the determination of the optimal processing parameters: grinding time of around 3.41 min and degree of grinding as *i* = 0.25. Multiple regression revealed temperature to be exerting the largest positive effect on the yield of the oil, and that over-grinding and pressure would affect efficiency in extraction adversely. Notably, pressure time was not statistically significant for oil yield, and process optimization should be directed mostly towards pressure and temperature, as suggested. The regression equations were satisfactory in goodness-of-fit and coefficients of determination (*R*^2^), mostly between 0.95, with satisfactory predictive ability and greatest statistical reliability.

The second key conclusion was presented by third-degree polynomial regressions that modeled correlations of degree of grinding and pressing time with certain process outputs such as cake density, temperature, energy, and shear stress. All models achieved perfect statistical fits (*R*^2^ = 1), i.e., there existed a very high level of experimental reproducibility and precise description of material behavior in tested ranges of parameters. Among the conditions tried, a degree of grinding of *i* = 0.25 was determined to yield best performance: minimum residual oil content in press cake, maximum yield stress, and maximum energy utilization, hence earning its technological superiority.

Comparative statistical analysis of oil quality indicated that all physicochemical parameters except two did not show any considerable difference between the traditional and experimental methods (e.g., density, viscosity, refractive index). Two of the parameters, saponification number and peroxide value, were improved significantly (*p* < 0.05) in the experimental method. Far more enlightening, though, were the changes in fatty acid composition: α-linolenic acid increased over tenfold (0.1% to 1.3%, *p* < 0.001), and oleic acid proportion also significantly increased (*p* < 0.01), implying greater oxidative stability and dietary quality. These statistically calculated results confirm that the new process method that emerges not only retains the inherent properties of safflower oil but also improves its functionality and compositional characteristics and presents a workable, resource-efficient solution for small and medium-sized oil manufacturers. The outcomes of this study have been successfully implemented in production, underscoring the reliability of the primary findings and conclusions. The newly developed equipment for safflower oil production is well-suited for use in small and medium-sized enterprises within the oil and fat industry.

## 5. Patents

Mursalykova Maygul Taurzhanovna, Kakimov Mukhtarbek Mukanovich, Kassenov Amirzhan Leonidovich, and Iskakov Bauyrzhan Myrzabekovich. Oil extraction screw press. For utility model patent No. 7977, filed 12 January 2023, and issued 21 April 2023.

Iskakov Bauyrzhan Myrzabekovich, Kakimov Mukhtarbek Mukanovich, Tokhtarov Zhaiyk Khamitovich, Mursalykova Maygul Taurzhanovna, and Sataeva Zhuldyz Isakovna. Centrifuge for deep purification of vegetable oils from mechanical impurities. For invention patent No. 36,262, filed 9 March 2022, and issued 15 September 2023.

## Figures and Tables

**Table 1:** Quadratic fit statistic.

*i*	*a* (*x*^2^ Coefficient)	*b* (x Coefficient)	*c* (Constant)	*R* ^2^
1	−0.00025	0.00164	0.008962	0.9097
0.5	−0.000425	0.00338	0.005431	0.9868
0.25	−0.0008	0.00616	0.0044	0.8826
0.125	−0.000625	0.0042	0.002031	0.995

**Table 2:** Comparison model fit table.

Degree of Grinding *i*	*R* ^2^	Fit Quality
0.25	0.9776	Excellent (best)
0.125	0.9678	Very good
0.5	0.9574	Very good
1	0.9504	Good

**Table 3:** Summary of model fit quality for different degrees of grinding based on energy consumption trends over time.

Degree of Grinding *i*	*R* ^2^	Fit Quality	Notes
1	1	Perfect	Slightly increasing energy with time
0.5	1	Perfect	Essentially quadratic trend
0.25	1	Perfect	Decreasing energy with time
0.125	1	Perfect	Nearly the same as for *i* = 0.25

**Table 4:** Effect of degree of grinding on compaction behavior and structural characteristics during pressing.

Degree of Grinding *i*	*R* ^2^	Fit Quality	Interpretation Highlights
0.25	1	Perfect	High peak density, slight drop at long *τ*
0.5	1	Perfect	Stable and dense structure
1	1	Perfect	Coarser texture, slower compaction
0.125	1	Perfect	Over-grinding leads to structural degradation

**Table 5:** Regression fit and mechanical behavior insights of safflower seed cake at varying degrees of grinding.

Degree of Grinding *i*	*R* ^2^	Fit Quality	Behavior Insight
0.25	1	Perfect	High shear strength, slight drop over time
0.5	1	Perfect	Stable mechanical behavior
1	1	Perfect	Lower cohesion, moderate decrease over time
0.125	1	Perfect	Over-fragmented, weakens rapidly during press

**Table 6:** Regression fit and thermal trend insights of safflower seed cake at varying degrees of grinding.

Degree of Grinding *i*	*R* ^2^	Fit Quality	Thermal Trend Insight
0.25	1	Perfect	Slight increase, then decline, stable peak
0.5	1	Perfect	Similar to that for *i* = 0.25, slightly cooler
1	1	Perfect	Lower temperatures, smooth thermal curve
0.125	1	Perfect	Continuous temperature drop due to overgrinding

**Table 7:** Physicochemical parameters of safflower oil.

No.	Parameter	Conventional Screw Press	Experimental Screw Press
1	Density (g/mL)	0.922 ± 0.190 *	0.917 ± 0.193 *
2	Specific weight	0.927 ± 0.194 *	0.960 ± 0.1 *
3	Refractive index	1.443 ± 0.243 *	1.464 ± 0.245 *
4	Viscosity (cP)	45.4 ± 1.35 *	45.9 ± 1.395 *
5	Acid number (mg KOH/g)	1.05 ± 0.209 *	1.063 ± 0.20 *
6	Iodine number (g in I_2_/100 g)	143.17 ± 2.42 *	144.13 ± 2.45 *
7	Peroxide number (mol/kg)	8.04 ± 0.54 *	7.61 ± 0.55 *
8	Saponification number (mg KOH/g)	159.3 ± 2.54 *	161.6 ± 2.59 *

* Values are expressed as arithmetic mean ± standard deviation.

**Table 8:** Statistical comparison of physicochemical parameters of safflower oil.

No.	Parameter	Conventional Screw Press (mean ± SD)	Experimental Screw Press (mean ± SD)	*p*-Value
1	Density (g/mL)	0.922 ± 0.190	0.917 ± 0.193	0.923
2	Specific weight	0.927 ± 0.194	0.960 ± 0.100	0.374
3	Refractive index	1.443 ± 0.243	1.464 ± 0.245	0.841
4	Viscosity (cP)	45.40 ± 1.350	45.90 ± 1.395	0.569
5	Acid number (mg KOH/g)	1.050 ± 0.209	1.063 ± 0.200	0.851
6	Iodine number (g I_2_/100 g)	143.17 ± 2.420	144.13 ± 2.450	0.052
7	Peroxide number (mol/kg)	8.040 ± 0.540	7.610 ± 0.550	0.043
8	Saponification number (mg KOH/g)	159.30 ± 2.540	161.60 ± 2.590	0.020

**Table 9:** Comparative indicators of fatty acid composition of safflower oil.

Lipid Number	Common Name	Mass Fraction of Fatty Acid (% of the Total Fatty Acids)	
		Conventional Screw Press	Experimental Screw Press
C_14:0_	Myristic acid	0.1 ± 0.06 *	0.2 ± 0.221 *
C_16:0_	Palmitic acid	6.3 ± 0.503 *	6.9 ± 0.563 *
C_16:1_	Palmitoleic acid	4.8 ± 0.449 *	5.0 ± 0.46 *
C_18:0_	Stearic acid	2.1 ± 0.301 *	2.0 ± 0.291 *
C_18:1_	Oleic acid	6.3 ± 0.506 *	7.9 ± 0.568 *
C_18:2_	Linoleic acid	84.2 ± 1.871 *	85.3 ± 1.187 *
C_18:3_	α-Linolenic acid	0.1 ± 0.062 *	1.3 ± 0.225 *
C_20:0_	Arachidic acid	0.5 ± 0.130 *	0.9 ± 0.184 *
Ʃ C_20:1_	Eicosenoic acids	0.2 ± 0.089 *	0.25 ± 0.101 *
C_22:0_	Behenic acid	0.4 ± 0.113 *	0.28 ± 0.108 *
Ʃ C_22:1_	Erucic acids	1.5 ± 0.256 *	2.7 ± 0.327 *

* Values are expressed as arithmetic mean ± standard deviation.

**Table 10:** Fatty acid composition of safflower oil produced by conventional and experimental methods.

Fatty Acid	Conventional (%) ± SD	Experimental (%) ± SD	*t*-Value	*p*-Value
C_14:0_ Myristic	0.1 ± 0.06	0.2 ± 0.221	≈−0.71	>0.5
C_16:0_ Palmitic	6.3 ± 0.503	6.9 ± 0.563	≈−1.77	~0.12
C_16:1_ Palmitoleic	4.8 ± 0.449	5.0 ± 0.460	≈−0.61	>0.5
C_18:0_ Stearic	2.1 ± 0.301	2.0 ± 0.291	≈0.39	>0.5
C_18:1_ Oleic	6.3 ± 0.506	7.9 ± 0.568	≈−4.79	<0.01
C_18:2_ Linoleic	84.2 ± 1.871	85.3 ± 1.187	≈−1.39	~0.20
C_18:3_ α-Linolenic	0.1 ± 0.062	1.3 ± 0.225	≈−11.75	<0.001
C_20:0_ Arachidic	0.5 ± 0.130	0.9 ± 0.184	≈−3.49	<0.02
Ʃ C_20:1_ Eicosenoic	0.2 ± 0.089	0.25 ± 0.101	≈−0.86	>0.4
C_22:0_ Behenic	0.4 ± 0.113	0.28 ± 0.108	≈2.01	~0.09
Ʃ C_22:1_ Erucic	1.5 ± 0.256	2.7 ± 0.327	≈−6.46	<0.001

## Data Availability

Data are contained within the article.
